# Role of sleep duration and sleep-related problems in the metabolic syndrome among children and adolescents

**DOI:** 10.1186/s13052-018-0451-7

**Published:** 2018-01-15

**Authors:** Leonardo Pulido-Arjona, Jorge Enrique Correa-Bautista, Cesar Agostinis-Sobrinho, Jorge Mota, Rute Santos, María Correa-Rodríguez, Antonio Garcia-Hermoso, Robinson Ramírez-Vélez

**Affiliations:** 1 Center of Studies in Physical Activity Measurements, School of Medicine and Health Sciences, Universidad del Rosario, Bogotá D.C, Colombia; 2 Research Center in Physical Activity, Health, and Leisure, Faculty of Sport, University of Porto, Porto, Portugal; 3 Early Start Research Institute, Faculty of Social Sciences, School of Education, University of Wollongong, Wollongong, Australia; 4 Faculty of Health Sciences, University of Granada, Granada, Spain; 5 Physical Activity, Sport and Health Sciences Laboratory, Faculty of Medical Sciences, Universidad de Santiago de Chile, USACH, Santiago, Chile

**Keywords:** Sleep quality, Cardiometabolic risk, Childhood obesity

## Abstract

**Background:**

There is increasing recognition that sleep is a risk factor for metabolic syndrome (MetS). The aim of the present study was to analyze the relationship between self-reported sleep duration, sleep-related problems and the presence of MetS in children and adolescents from Bogotá, D.C., Colombia.

**Methods:**

This is a cross-sectional analysis from the FUPRECOL study (2014–15). Participants included 2779 (54.2% girls) youth from Bogota (Colombia). MetS was defined as the presence of ≥3 of the metabolic abnormalities (hyperglycemia, hypertriglyceridemia, low high-density lipoprotein cholesterol [HDL-c], hypertension, and increased waist circumference) according to the criteria of de Ferranti/Magge and colleges. Self-reported sleep duration and sleep-related problems were assessed with the BEARS questionnaire.

**Results:**

Logistic regression analysis showed that boys who meet recommended duration of sleep had a decreased risk of elevated blood glucose levels (Odds Ratio [OR] = 0.71, 95%CI [0.40–0.94]; *p* = 0.031) compared to boys who have short-long sleep duration. Also, compared to young without sleep problems, excessive sleepiness during the day was related to low HDL-c levels in boys (OR = 1.36, 95%CI [1.02–1.83]; *p* = 0.036) and high triglyceride levels in girls (OR = 1.28, 95%CI [1.01–1.63]; *p* = 0.045). Girls with irregular sleep patterns had decreased HDL-c levels (OR = 0.71, 95%CI [0.55–0.91]; *p* = 0.009).

**Conclusions:**

Recommended sleep duration was associated with a decreased risk of elevated fasting glucose levels in boys, and sleep problems was related to lower HDL-c in girls and higher triglyceride levels in boys. These findings suggested the clinical importance of improving sleep hygiene to reduce metabolic risk factors in children and adolescents.

## Background

Insufficient sleep has been postulated as predictors of emerging cardiometabolic risk markers [1]. Sleep duration and sleep quality have been associated with obesity [2], diabetes [3], and hypertension [4], strong and independent indicators of cardiovascular disease risk.

Sleep duration has recently been recognized as a risk factor for metabolic syndrome (MetS) [5]. To date, most studies examining the relationships between sleep duration and MetS or its components (waist circumference percentile, triglyceridemia, HDL-c, blood pressure, and fasting glucose) have been conducted in adults, and little is known about these relationships in the pediatric population. In addition, the limited research has yielded inconsistent findings [6–13]. Evidence for an association with sleep duration appears more consistent for fasting glucose, suggesting that both short and long sleep duration might play a determinant role MetS in children and youth [7, 9].

Similarly, only few studies have investigating the relationships between sleep-related problems and MetS, as most research has focused on sleep duration [14, 15]. For example, Kuula et al. (2016) reported that poor sleep duration during the transition to early adolescence was related to an atherogenic lipid profile, and with this effect is less prominent in boys [14]. Likewise, long sleeper during the day was associated with low high-density lipoprotein cholesterol (HDL-c) in girls (but not in boys) in a large sample of children aged 11–12 years from The Netherlands [15]. Regarding sleep quality, poor sleep has also been associated with high diastolic blood pressure and waist circumference (but not with other MetS components) in a cohort of 135 Brazilian children and adolescents [16]. Therefore, because previous research reported inconsistent results and considering that the sample sizes of these studies were relatively small, no conclusion can be drawn as yet.

Identifying the impact of sleep duration and sleep-related problems on metabolic risk factors in children and adolescents will enable the establishment of early interventions for pediatric sleep problems. Due to the absence of sufficient conclusive evidence regarding the effect of sleep characteristics on metabolic risk factors in pediatric populations, the aim of the present study was to analyze the relationship between self-reported sleep duration, sleep-related problems and the presence of MetS in children and adolescents from Bogotá, D.C., Colombia.

## Methods

### Study design and participants

During the 2014–2015 school years (academic year), we examined a cross-sectional component of the FUPRECOL study [17] (in spanish: *Asociación de la fuerza prensil con manifestaciones de riesgo cardiovascular tempranas en niños y adolescentes colombianos).* The sample consisted of children and adolescents (*n* = 4000 boys and *n* = 4000 girls) aged 9–17.9 years. Blood sampling was randomly performed in one third of the recruited subjects (*n* = 2900). In this subgroup, valid data were available for 2779 apparently healthy children and adolescents without any physical or mental medical condition diagnosis (54.2% girls) on self-reported hours of sleep and all components included in the biochemical samples. Participants belong to low and middle socioeconomic status (1–3 defined by the Colombian government). Children and adolescents were enrolled in public elementary and high schools (grades 5 through 11) from the capital district of Bogotá in a municipality in the Cundinamarca Department in the Andean region. A convenience sample of volunteers was included and grouped by sex and age in 1-year increments (a total of nine groups).

### Anthropometric measurements

Participants were instructed to wear shorts and a t-shirt for the physical examination. They were also required to remove all jewelry and other metal objects. Once the subjects were barefoot and in their underwear, their body weight (kg) was measured using an electric scale (Model Tanita® BC-418® Tokyo, Japan) with a range of 0–200 kg and accurate to within 100 g. Height was measured with a portable stadiometer with a precision of 0.1 mm and a range of 0–2.50 m (Seca® 206, Hamburg, Germany). Body mass index (BMI) was calculated as weight (kg)/height (m^2^), and BMI-z score was calculated using the World Health Organization Growth Reference 2007 [18]. The waist circumference (WC) (cm) was measured as the narrowest point between the lower costal border and the iliac crest. When this point was not evident, it was measured at the midpoint between the last rib and the iliac crest, using a metal tape measure (Lufkin W606 PM®, Parsippany, New Jersey, USA), in accordance with the International Society for the Advancement of Kinanthropometry guidelines [19]. The evaluation process was carried out by a team of professionals (four physical activity Master’s degree students and physiotherapist professors) with extensive experience in anthropometric measurement. Two percent of the sample was measured twice in order to ensure measurement consistency. The technical error of measurement was less than 2% for all anthropometric variables [17].

### Blood pressure

Systolic (SBP) and diastolic blood pressure (DBP) was taken on the left arm at the heart level with an automatic device (Riester Ri-Champion model, Jungingen, Germany) while the participants were sitting still. The blood pressure monitor cuff was placed two to three finger-widths above the bend of the arm, and a 2-min pause was allowed between the first and second measurements taken with a standard cuff for an arm circumference of 17–22 cm.

### Blood sampling and analysis

After the subjects had fasted for 10–12 h, blood samples were obtained by capillary sampling between 6:00 a.m. and 8:00 a.m. Participants were asked not to engage in prolonged exercise in the 24 h prior to testing. The biochemical profile included the following: (i) HDL-c, (ii) triglycerides, (iii) total cholesterol, and (iv) fasting blood glucose, assayed by enzymatic colorimetric methods. The inter-assay reproducibility (coefficient of variation) was determined from 16 replicate analyses of eight plasma pools over a period of 15 days. The percentages obtained were 2.6% for triglycerides, 2.0% for total cholesterol, 3.2% for HDL-c, 3.6%, and 1.5% for fasting glucose.

### Metabolic syndrome diagnosis

MetS was defined in accordance with the updated harmonized criteria of de Ferranti et al. [20] and Magge et al. [21] based on age/gender/height criteria. Participants were considered to have MetS if they showed three or more of the following: (1) abdominal obesity (WC >75th percentile), (2) hypertriglyceridemia (≥100 g/dL), (3) low HDL-c (< 50 mg/dL), (4) high blood pressure (>90th percentile), and (5) high fasting glucose (≥110 mg/dL).

### Cardiorespiratory fitness assessment

Cardiorespiratory fitness was measured using the 20-m shuttle run test as previously described by Léger [22]. Results were recorded to the nearest stage completed. The Léger equation was used to determine VO_2_peak (ml·kg·min^− 1^) in each participant. The test was performed in according to the standardized protocol and the detailed description of this test can be found elsewhere [23]. The reproducibility (R) of our data was *R* = 0.84. Intra-rater reliability was assessed by determining the intra-class correlation coefficient (ICC = 0.96, CI 95% 0.95–0.97). We used this parameter as covariate due to its relationship with sleep quality [24].

### Sexual maturity

Participants self-assessed their pubertal stage of secondary sex characteristics (breast and pubic hair development for girls, genital and pubic hair development for boys; ranging from stage I to V), according to the criteria of Tanner and Whitehouse [25].

### Self-reported duration and sleep problems

Participants self-reported their sleep duration as the average nightly sleep time on weekdays and weekend days by answering the following questions: How long do you generally sleep on school days? How long do you generally sleep on the weekend? According to the recommendations of the National Sleep Foundation [26] sufficient sleep was considered young who slept between 9 to 11 h or 8 to 10 in children and adolescents, respectively. Short and long sleep duration were defined according to current recommendation as 8 to 9 h, and 10 to 11 h sleep per night, respectively [26].

Additionally, we used the BEARS questionnaire to recognize sleep-related problems (sleep quality) [27]. This screening tool prompts the professional care to ask scholar children an initial screening question about possible problems in each domain, eliciting a yes or no response. This instrument includes a set of basic questions that evaluate sleep-related areas such as (i) bedtime problems, including difficulty going to bed and falling asleep; (ii) excessive daytime sleepiness, which includes behaviors typically associated with daytime somnolence in children; (iii) awakenings during the night; (iv) regularity and duration of sleep; (v) and snoring. Because sleep-related problems were self-reported, and subjects are usually not aware of snoring, this item was not assessed. The responses were restricted to the last 7 days, and the frequency option was categorized as ‘yes’ or ‘no’. This questionnaire has shown adequate validity and reliability in young populations [27, 28]. On the website of the Spanish Association of Primary Care Pediatrics, it is provided the Spanish translation of BEARS that was used in this study (http://www.aepap.org/gtsiaepap/). Recently, Bastida-Pozuelo et al. [28] analysed the validity of the BEARS questionnaire evaluating how well it performs to distinguish young populations with specific sleep problems using the Children Sleep Habits Questionnaire scores as the gold standard. These authors shown the participants aged 2–16 years who, according to the BEARS, had a sleep problem obtained scores on the Sleep Habits Questionnaire scores-related subscales significantly higher than children and adolescents who did not have a sleep problem (all Ps < 0.05). Nevertheless, considering the lack of psychometric properties in the Colombian population, a reliability analysis was conducted, revealing internal consistency results (Cronbach’s alpha) of 0.75 (min 0.70 to max 0.76). Furthermore, in a sub-sample of 229 school-aged children (mean age 12.8 ± 2.4 years, weight 46.2 ± 12.4 kg, height 1.50 ± 0.1 m, and BMI 19.9 ± 3.1 kg/m^2^) and a 7-day period between each test, test-retest (Kappa index) reproducibility values of 0.665 were obtained for the four questions of the BEARS questionnaire.

The surveys were administered to school-aged children in groups of 20–50 participants in classrooms in order to maintain privacy and to give the students free choice to participate and in the presence of at least two qualified researchers. The questionnaires were completed in approximately 3–5 min. Prior to questionnaire administration, the necessary guidelines were provided for their completion, highlighting the need to carefully read the items and the importance of sincerity and confidentiality when answering them.

### Ethics statement

The FRUPECOL study was conducted in accordance with the Helsinki Declaration for Human Studies and approved by the Colombian Data Protection Authority (Resolution 008430/1993 Ministry of Health) and the Review Committee for Research on Human Subjects at the University of Rosario (Code N° CEI-ABN026-000262). All participants were informed of the study’s goals, and written informed consent was obtained from participants and their parents or legal guardians.

### Statistical analysis

The continuous variables were expressed as mean (SD), and the categorical variables were expressed as frequencies. To assess statistical normality, the Kolmogorov-Smirnov test was used. Because of their skewed distribution, the following variables were log-transformed before analysis: WC, HDL-c, fasting glucose, triglycerides, SBP and DBP. To aid interpretation, data were back-transformed from the log scale for presentation in the results.

Differences between the sexes were analyzed by Student’s *t*-test or chi-square test. ANCOVA analysis was used to evaluate differences in sleep duration and sleep problems categories for each MetS components. We performed logistic regression order to determine the risk of having MetS or the altered levels of each of the MetS components taking as reference group participants who slept short-long duration or have sleep problems, respectively. Results were expressed as odds ratios (ORs) and 95% CI. Analyses were adjusted by age, sexual maturity, body mass index (except for waist circumference), and cardiorespiratory fitness. SPSS version 22.0 (SPSS Inc., Chicago, IL) software was used for statistical analyses. A *p* value 0.05 denoted statistical significance.

## Results

Table 1 shows the characteristics of the participants, by sex. The mean age of the sample was 13.21 (2.2) years. Boys were heavier and taller and had better cardiorespiratory fitness than girls. In contrast, girls had higher BMI values, higher triglyceride levels, and a longer average sleep duration at the weekend than boys. The average sleep duration over the entire week was 8.11 (1.61) hours per night. The prevalence of the MetS was 9.65% and was statistically significantly higher in girls than in boys (*p* < 0.05).

**Table 1:** Characteristics of the sample, by sex

Characteristics	Boys (*n* = 1272)	Girls (*n* = 1507)	*P* value
Anthropometrics			
Age, (years)	13.26 (2.19)	13.17 (2.21)	0.294
Weight, (kg)	47.55 (12.55)	46.16 (10.67)	0.002
Height, (cm)	155.55 (13.87)	150.64 (9.54)	< 0.001
Body mass index, (kg/m^2^)	19.33 (2.90)	20.11 (3.18)	< 0.001
Body mass index, (z-score)	0.39 (0.91)	0.43 (0.78)	< 0.001
Waist circumference, (cm)	65.69 (7.42)	63.70 (7.42)	< 0.001
Sexual maturity			
Prepuberty, n (%)	85 (6.68)	78 (5.17)	
Puberty, n (%)	650 (51.11)	734 (48.77)	0.157
Postpuberty, n (%)	537 (42.21)	696 (46.18)	
Blood sampling			
HDL-c, (mg/dL)	46.91 (12.36)	47.49 (12.23)	0.215
Triglycerides, (mg/dL)	85.19 (39.07)	96.53 (54.73)	< 0.001
Fasting blood glucose, (mg/dL)	83.45 (15.82)	81.52 (15.72)	0.001
Systolic blood pressure, (mmHg)	113.39 (14.04)	110.30 (12.36)	< 0.001
Diastolic blood pressure, (mmHg)	68.19 (9.31)	68.45 (8.59)	0.443
Cardiorespiratory fitness, (ml.kg.min^−1^)	50.13 (3.66)	35.55 (2.79)	< 0.001
Sleep duration			
Week days, hours/night	8.11 (1.61)	8.09 (1.69)	0.811
Weekend, hours/night	9.63 (1.85)	9.82 (1.91)	0.010
Short-long duration^a^	479 (37.7)	939 (62.3)	0.102
Sleep patterns			
Short sleep duration, n (%)	900 (70.48)	1055 (69.78)	0.686
Bedtime problems, n (%)	292 (22.87)	374 (24.74)	0.249
Excessive day sleepiness, n (%)	396 (31.01)	673 (44.51)	< 0.001
Awakenings during the night, n (%)	248 (19.42)	320 (21.16)	0.255
Regularity and duration of sleep, n (%)	407 (31.87)	611 (40.41)	< 0.001
Metabolic syndrome (abnormal values)			
High waist circumference, n (%)^b^	55 (4.38)	50 (3.34)	0.155
Low HDL-c levels, n (%)	833 (65.23)	978 (64.68)	0.762
High triglycerides levels, n (%)	278 (21.77)	497 (32.87)	< 0.001
High fasting blood glucose, n (%)	67 (5.25)	68 (4.50)	0.358
High systolic blood pressure, n (%)^c^	201 (16.22)	196 (13.17)	0.025
High diastolic blood pressure, n (%)^c^	130 (10.49)	165 (11.09)	0.618
Components metabolic syndrome, n (%)^d^			
0	280 (22.16)	317 (21.03)	0.017
1	571 (44.87)	615 (40.94)	
2	308 (24.12)	419 (27.71)	
3	82 (6.42)	132 (8.73)	
4	26 (2.04)	19 (1.26)	
5	5 (0.39)	5 (0.33)	

Values are means (SD), except for categorical data n (%)

^a^ According to the National Sleep Foundation cut-off [25]; ^b^ (*n* = 2756) (Boys = 1257 Girls = 1499); ^c^ (*n* = 2727) (Boys = 1239 Girls = 1488); ^d^ Three or more components according to Ferranti et al. criteria

Table 2 shows the metabolic parameters according to sleep duration and sleep-related problems. In boys, diastolic blood pressure values were significantly higher in those without bedtime problems, and triglyceride values were significantly higher in those with excessive daytime sleepiness. In girls, systolic blood pressure was significantly higher in the absence of bedtime problems.

**Table 2:** Mean differences of metabolic parameters according sleep duration and sleep quality, by sex

	Boys			Girls		
Sleep duration	Recommended duration^a^	Short-Long duration^a^	*P* value	Recommended duration^a^	Short-Long duration^a^	*P* value
Waist circumference, (cm)	65.91 (7.58)	65.04 (7.38)	0.545	63.77 (7.18)	63.12 (7.62)	0.393
HDL-c, (mg/dL)	46.50 (12.41)	47.49 (12.52)	0.652	48.11 (12.75)	47.50 (11.62)	0.438
Triglycerides, (mg/dL)	85.78 (38.87)	83.70 (36.91)	0.463	96.03 (53.37)	95.31 (43.76)	0.722
Fasting blood glucose, (mg/dL)	84.76 (15.79)	83.70 (36.91)	0.191	82.07 (16.16)	82.44 (16.20)	0.905
Systolic blood pressure, (mmHg)	113.72 (14.17)	114.00 (13.78)	0.427	110.70 (12.20)	110.89 (12.38)	0.801
Diastolic blood pressure, (mmHg)	68.29 (9.60)	68.32 (9.34)	0.618	68.67 (8.81)	68.49 (8.94)	0.899
Bedtime problems	Yes	No	P value	Yes	No	P value
Waist circumference, (cm)	65.75 (0.45)	65.41 (0.48)	0.601	63.40 (0.41)	63.69 (0.32)	0.693
HDL-c, (mg/dL)	45.68 (0.89)	46.30 (0.97)	0.653	47.00 (0.87)	48.06 (0.69)	0.370
Triglycerides, (mg/dL)	90.86 (2.89)	86.41 (3.13)	0.496	95.64 (3.98)	97.67 (3.17)	0.948
Fasting blood glucose, (mg/dL)	84.97 (1.23)	85.93 (1.33)	0.602	80.12 (1.13)	81.47 (0.90)	0.463
Systolic blood pressure, (mmHg)	114.22 (1.09)	114.82 (1.17)	0.738	108.82 (0.87)	111.89 (0.69)	0.007
Diastolic blood pressure, (mmHg)	67.44 (0.72)	69.70 (0.77)	0.030	67.50 (0.61)	68.91 (0.48)	0.088
Excessive day sleepiness						
Waist circumference, (cm)	65.77 (0.55)	65.39 (0.36)	0.693	63.11 (0.35)	63.98 (0.38)	0.090
HDL-c, (mg/dL)	46.25 (1.10)	45.73 (0.73)	0.808	48.07 (0.76)	46.99 (0.81)	0.185
Triglycerides, (mg/dL)	93.41 (3.54)	83.86 (2.35)	0.032	93.27 (3.48)	100.04 (3.71)	0.135
Fasting blood glucose, (mg/dL)	85.81 (1.50)	85.09 (1.00)	0.575	79.98 (0.99)	81.62 (1.05)	0.208
Systolic blood pressure, (mmHg)	114.64 (1.33)	114.40 (0.88)	0.741	110.44 (0.76)	110.27 (0.81)	0.743
Diastolic blood pressure, (mmHg)	69.28 (0.88)	67.87 (0.59)	0.153	68.12 (0.53)	68.28 (0.57)	0.821
Awakenings during the night						
Waist circumference, (cm)	66.05 (0.57)	65.10 (0.33)	0.185	63.46 (0.44)	63.63 (0.28)	0.615
HDL-c, (mg/dL)	45.20 (1.14)	46.77 (0.66)	0.129	48.26 (0.93)	46.80 (0.60)	0.175
Triglycerides, (mg/dL)	90.81 (3.68)	86.46 (2.14)	0.420	98.26 (4.26)	95.05 (2.77)	0.129
Fasting blood glucose, (mg/dL)	85.50 (1.56)	85.39 (0.91)	0.916	80.44 (1.21)	81.15 (0.78)	0.593
Systolic blood pressure, (mmHg)	115.04 (1.38)	114 (0.81)	0.509	111.20 (0.93)	109.51 (0.60)	0.199
Diastolic blood pressure, (mmHg)	69.05 (0.91)	68.09 (0.53)	0.342	68.56 (0.65)	67.84 (0.42)	0.422
Regularity and duration of sleep						
Waist circumference, (cm)	64.97 (0.51)	66.19 (0.42)	0.127	63.58 (0.33)	63.51 (0.40)	0.953
HDL-c, (mg/dL)	46.05 (1.02)	45.93 (0.83)	0.791	47.06 (0.70)	48.00 (0.85)	0.369
Triglycerides, (mg/dL)	87.21 (3.29)	90.06 (2.70)	0.631	97.90 (3.23)	95.40 (3.92)	0.557
Fasting blood glucose, (mg/dL)	85.66 (1.40)	85.23 (1.15)	0.828	81.91 (0.92)	79.69 (1.11)	0.126
Systolic blood pressure, (mmHg)	114.48 (1.23)	114.56 (1.02)	0.987	111.00 (0.70)	109.71 (0.86)	0.244
Diastolic blood pressure, (mmHg)	68.59 (0.81)	68.56 (0.67)	0.932	68.45 (0.49)	67.96 (0.60)	0.563

Data are means (SD). Analysis adjusted by age, sexual maturity, body mass index (except for waist circumference), and cardiorespiratory fitness. ^a^ According to the National Sleep Foundation cut-off [25]

Logistic regression analysis of sleep time, sleep-related problems, and the MetS components are shown in Table 3. Boys who slept recommended duration were at a decreased risk of higher fasting blood glucose (OR = 0.71, 95%CI [0.40–0.94], *p* = 0.031) compared to those who reported short-long duration. Also compared to young without sleep problems, those who reported excessive daytime sleepiness had lower HDL-c levels (OR = 1.36, 95% CI [1.02–1.83], *p* = 0.036). In girls, excessive daytime sleepiness also was associated with an increased risk of elevated triglyceride levels (OR = 1.28, 95%CI [1.01–1.63], *p* = 0.045), while an irregular sleep pattern was related to low HDL-c levels (OR = 0.71, 95%CI [0.55–0.91], *p* = 0.009).

**Table 3:** Odd ratios of metabolic parameters from sleep duration and sleep patterns, by sex

	Boys			Girls		
	OR	(95% CI)	*P* value	OR	(95% CI)	*P* value
Sleep Duration						
High waist circumference	1.79	(0.77–4.18)	0.175	1.54	(0.76–3.12)	0.226
Low HDL	1.05	(0.80–1.39)	0.701	1.07	(0.84–1.36)	0.569
High triglycerides	0.93	(0.68–1.26)	0.634	1.03	(0.80–1.32)	0.815
High fasting blood glucose	0.71	(0.40–0.94)	0.031	1.26	(0.74–2.12)	0.392
High systolic blood pressure	1.12	(0.81–1.56)	0.482	1.09	(0.78–1.53)	0.607
High diastolic blood pressure	0.77	(0.51–1.16)	0.221	0.76	(0.54–1.09)	0.145
Presence metabolic syndrome	0.86	(0.54–1.36)	0.513	0.77	(0.53–1.13)	0.181
Bedtime problems						
High waist circumference	0.62	(0.19–2.01)	0.433	0.53	(0.24–1.32)	0.175
Low HDL	0.92	(0.65–1.30)	0.655	1.08	(0.80–1.44)	0.600
High triglycerides	1.29	(0.94–1.76)	0.112	1.00	(0.74–1.34)	0.987
High fasting blood glucose	0.76	(0.40–1.46)	0.424	1.51	(0.73–3.08)	0.259
High systolic blood pressure	1.07	(0.70–1.65)	0.732	1.11	(0.73–1.69)	0.608
High diastolic blood pressure	0.78	(0.48–1.26)	0.316	1.17	(0.75–1.82)	0.477
Presence metabolic syndrome	0.72	(0.42–1.25)	0.253	1.46	(0.89–2.39)	0.129
Excessive day sleepiness						
High waist circumference	1.04	(0.39–2.79)	0.926	0.88	(0.43–1.76)	0.719
Low HDL	1.36	(1.02–1.83)	0.036	0.99	(0.78–1.26)	0.977
High triglycerides	0.88	(0.64–1.23)	0.482	1.28	(1.01–1.63)	0.045
High fasting blood glucose	1.15	(0.63–2.10)	0.645	0.96	(0.55–1.65)	0.882
High systolic blood pressure	1.21	(0.83–1.77)	0.317	1.27	(0.90–1.80)	0.172
High diastolic blood pressure	1.00	(0.65–1.54)	0.979	1.24	(0.87–1.78)	0.229
Presence metabolic syndrome	1.46	(0.86–2.47)	0.151	1.24	(0.84–1.82)	0.262
Awakenings during the night						
High waist circumference	0.82	(0.27–2.49)	0.732	1.81	(0.80–4.09)	0.149
Low HDL	1.01	(0.70–1.44)	0.954	1.27	(0.94–1.72)	0.115
High triglycerides	1.10	(0.73–1.66)	0.631	1.00	(0.74–1.36)	0.966
High fasting blood glucose	1.89	(0.84–4.20)	0.119	0.77	(0.39–1.52)	0.465
High systolic blood pressure	0.85	(0.55–1.32)	0.495	0.70	(0.46–1.07)	0.105
High diastolic blood pressure	0.83	(0.50–1.38)	0.491	0.97	(0.62–1.52)	0.902
Presence metabolic syndrome	1.34	(0.73–2.48)	0.337	0.97	(0.60–1.59)	0.927
Regularity and duration of sleep						
High waist circumference	0.47	(0.19–1.18)	0.111	0.55	(0.25–1.18)	0.126
Low HDL	0.96	(0.71–1.29)	0.820	0.71	(0.55–0.91)	0.009
High triglycerides	0.97	(0.69–1.35)	0.864	0.98	(0.76–1.26)	0.891
High fasting blood glucose	0.65	(0.37–1.14)	0.135	1.01	(0.57–1.78)	0.972
High systolic blood pressure	0.98	(0.68–1.41)	0.919	1.05	(0.73–1.51)	0.766
High diastolic blood pressure	0.89	(0.58–1.36)	0.603	0.86	(0.60–1.25)	0.457
Presence metabolic syndrome	0.87	(0.54–1.41)	0.579	1.07	(0.72–1.59)	0.719

Reference group (Odds Ratio = 1.0): short-long duration sleep duration or non-sleep problem. Analysis adjusted by age, sexual maturity, body mass index (except for waist circumference), and cardiorespiratory fitness

## Discussion

Sleep duration and sleep patterns have been proposed as potential risk factors contributing to increased metabolic risk at an early age [29]. This study investigated the relationships between sleep time and sleep-related problems and metabolic risk factors in a cohort of 2779 children and adolescents, suggesting a determinant role of sleep in components of MetS. To our knowledge, our study comprised the largest cohort of Latin American children assessed to date.

In recent years, there has been an increasing number of studies about sleep duration and its impact on metabolic health during childhood and adolescence [1, 5]. With regard to glucose and insulin homeostasis, accumulating evidence suggests that sleep time may play a relevant role [30]. However, most published studies examining the association between sleep duration and glycaemia have been conducted in adults, and the limited data available in children and adolescents has demonstrated contradictory findings [6–9]. In this regard, a recent systematic review in young people (aged 0–19 years) suggested that insufficient sleep duration might has a negative effect on glucose homeostasis [31]. In the present study and confirming above-mentioned results, we identified that recommended duration was associated with decreased levels of fasting glucose in boys. Sleep duration was also recently positively associated with insulin resistance in a cohort of obese children and adolescents [32], suggesting that children 10–13 years old who slept < 9 h per day were more likely to have higher insulin and HOMA-IR levels and lower HDL-c levels compared with subjects who slept 9–10 h/ per day and > 10 h per day. Interestingly, previous published studies in young population found U-shaped relationships between total sleep time and glucose levels [7, 9]. Thus, in the Cleveland Children’s Sleep and Health Cohort Study, adolescents (aged 16–19 years) who slept 7.75 h per night had a lower risk of elevated fasting blood glucose and those with short sleep duration (5.0 h) or long sleep duration (10.5 h) had HOMA levels that were about 25% [9]. The lack of relationship between shorter sleep duration and glucose levels reported in our study may be explained by the different confounding variables included in the analysis and the data on sleep reported as the mean of the days. In this context, experimental studies have identified that sleep restriction and extension exert an effect on insulin sensitivity, but the underlying mechanistic links have not been well determined [33, 34]. Based on our results and previous research, we may speculate that excessive amounts of sleep contribute to increased fasting glucose levels in children and adolescents.

On the other hand, we failed to find significant associations between sleep duration and other MetS components, such as waist circumference, HDL-c, triglycerides, and blood pressure levels, after adjusting for potential confounders, which might be due to self-reported data. Conflicting and sparse data have been encountered on the impact of sleep time on these components of MetS in children and adolescents [10, 12, 13, 35]. The discrepancies identified across different studies could be explained in part by the following reasons. Remarkably, most studies investigating these associations have been conducted in overweight and obese children and adolescents [7, 13, 16, 35]. Note that our study population included underweight, normal-weight, overweight, and obese subjects. Furthermore, in this study, considering the recommendations of the National Sleep Foundation, 9 h per night was taken as the ideal cut-off point; however, not all studies above-mentioned have used the same cut-off point.

On the other hand, we found that 39.5% of children and adolescents obtained short or long duration of sleep on a regular basis. Likewise, data from the 2016 United States Youth Risk Behavior Survey reported that the prevalence of obtaining 8 or more hours of sleep was 27.3% [36]. Moreover, in line with a previous published study [37], excessive daytime sleepiness and lack of regular sleep were the most prevalent sleep problems in the Latin American pediatric population (44.51% and 40.41%, respectively). In addition, we observed that girls slept less than boys and presented a higher prevalence of sleep problems (assessed with the Pittsburgh Sleep Quality Index); similar results were found in a cohort of Brazilian overweight or obese children and adolescents (aged 5–18 years) [16].

Because sleep is a multidimensional construct, in addition to sleep duration, it was especially relevant to identify whether sleep quality was associated with metabolic risk factors. To date, evidence regarding the effect of sleep quality on metabolic risk is lacking, because the majority of studies conducted in the pediatric population have focused on sleep duration [14, 15]. We found that excessive sleepiness during the day was related to lower levels of HDL-c in boys (OR = 1.36, 95%CI [1.02–1.83], *p* = 0.036), and higher levels of triglycerides in girls (OR = 1.28, 95% CI [1.01–1.63], *p* = 0.045). In agreement with our findings, Berentzen et al. (2016) reported that in a cohort of 1481 children, subjects who felt sleepy during the day also had lower HDL-c levels [14]. Similarly, a longitudinal study showed that poor sleep quality was linked to higher triglyceride levels [14]. Interestingly, in our study, irregular sleep patterns were linked to a protective HDL-c levels in girls (OR = 0.71, 95%CI [0.55–0.91], *p* = 0.009). Taken together, these lines of evidence indicate that sleep patterns may have an importance influence on serum lipids levels. However, due to the limited evidence regarding sleep quality and components of the MetS, further research is needed to confirm our preliminary results.

The present study indicates that sleep duration and sleep-related problems are not associated with MetS. Previous data in different cohorts of children and adolescents have showed conflicting findings, mostly negative [1, 12–14, 16, 38]. Note that in data from 1187 adolescents of the Korean National Health and Nutrition Examination Survey (KNHANES) IV, although there were relationships between sleep duration and MetS components, no association was identified with the MetS [12]. The contrasting relationships observed in our study may be explained by a lack of statistical power due to the low prevalence of MetS (9.65%).

There are some limitations to the present study. Firstly, the cross-sectional design does not allow us to make cause effect inferences between sleep duration and MetS components. Thus, additional prospectively and intervention are necessary to determine causal links. Secondly, because sleep measurements were based on the self-report method instead of objective polysomnography or accelerometry, limitations in terms of reliability and validity are inherent in the methodology. Nevertheless, objective measures of sleep are often not suitable for large epidemiologic studies due to its high costs. Moreover, a rather high correlation between subjective and objective sleep indices in children has been identified [39]. Also, youths were asked to express the data on sleep as the mean of more days, which could influence on the reliability of the data.

Similarly, there are several confounders that may influence and modulate the association between sleep-disordered breathing and MetS. Variables such as physical activity, diet, socioeconomic status, menstrual cycle and race are likely important modulators of this association. On the other hand, snoring as another important risk factors for developing MetS at early ages. In this context, in the present study, this item was not assessed because sleep-related problems were self-reported by participants. However, the extent to which each of metabolic abnormalities factors interact with snoring and contributes to the development of metabolic consequences in snoring youth population has yet to be delineated [40]. Finally, it is important to highlight that the BEARS questionnaire has been postulated as a useful pediatric sleep screening tool for identifying sleep problems [27] and recently a previous article supports the concurrent and reliability validity of the Spanish translation of the BEARS to detect sleep problems in pediatric nursing assessments [28, 41].

On the other hand, the main strength of this study is the large representative sample that allowed us to provide data on a national scale. Furthermore, highly standardized procedures were developed within the FRUPECOL study to avoid measurement bias. Moreover, information on age, sexual maturity, and cardiorespiratory fitness were considered as potential confounding variables.

## Conclusion

To the best of our knowledge, this is the first study to describe comprehensively the relationship between sleep duration and sleep related-problems and metabolic markers in Latin American children and adolescents. We report novel results on the relationship between sleep characteristics and elements of the MetS. Our study found that recommended sleep duration based on subjective parameters was associated with decreased fasting glucose levels, and poor sleep quality was related to lower HDL-c and higher triglyceride levels, suggesting the clinical importance of improving sleep hygiene to reduce metabolic risk factors in children and adolescents. In contrast, we could not find significant relationship in the rest of the parameters, which might be due to self-reported data. Future prospective and intervention studies with objective assessments are needed to establish a possible causal link between sleep patterns and metabolic risk factors.

## Abbreviations

BMI: Body mass index
DBP: Diastolic blood pressure
HDL-c: High-density lipoprotein cholesterol
LDL-c: Low high-density lipoprotein cholesterol
MetS: Metabolic syndrome
OR: Odds Ratio
SBP: Systolic blood
WC: Waist circumference
